# Modulatory Effect of 1,25-Dihydroxyvitamin D_**3**_ on IL1****β****-Induced RANKL, OPG, TNF****α****, and IL-6 Expression in Human Rheumatoid Synoviocyte MH7A

**DOI:** 10.1155/2013/160123

**Published:** 2013-11-18

**Authors:** Xiaoke Feng, Chengyin Lv, Fang Wang, Ke Gan, Miaojia Zhang, Wenfeng Tan

**Affiliations:** ^1^Department of Rheumatology, The First Affiliated Hospital of Nanjing Medical University, 300 Guangzhou Road, Nanjing, Jiangsu 210029, China; ^2^Department of Traditional Chinese Medicine, The First Affiliated Hospital of Nanjing Medical University, Nanjing, Jiangsu 210029, China; ^3^Department of Cardiology, The First Affiliated Hospital of Nanjing Medical University, Nanjing, Jiangsu 210029, China; ^4^Department of Internal Medicine in Traditional Chinese Medicine, Nanjing Traditional Chinese Medicine University, Nanjing, Jiangsu 210029, China

## Abstract

Receptor activator of nuclear factor **κ**B ligand (RANKL) plays a crucial role in the bone erosion of rheumatoid arthritis (RA) by prompting osteoclastogenesis. Considering that 1,25(OH)_2_D_3_ has been suggested as a potent inducer of RANKL expression, it should clarify whether vitamin D supplement could result in RANKL overexpression and thereby facilitate excessive osteoclastogenesis and bone resorption in RA. Here, we investigated modulatory effect of 1,25(OH)_2_D_3_ on the expression of RANKL and its decoy receptor osteoprotegerin (OPG) in an inflammatory condition of human rheumatoid synoviocyte MH7A. MH7A cells were stimulated with IL1**β** and then treated with different concentrations of 1,25(OH)_2_D_3_ for 48 h. A significantly elevated OPG/RANKL ratio and markedly decreased levels of IL-6 and TNF**β** mRNA expression in cells and IL-6 protein in supernatants were observed in IL1**β**-induced MH7A in the presence of 1,25(OH)_2_D_3_ compared with those in the absence of it. Osteoclast formation was obviously decreased when RAW264.7 cells were treated with both 1,25(OH)_2_D_3_ and IL1**β**. In summary, although it has a biological function to induce RANKL expression, 1,25(OH)_2_D_3_ could upregulate OPG/RANKL ratio and mediate anti-inflammatory action in an inflammatory milieu of synoviocyte, contributing to the inhibition of inflammation-induced osteoclastogenesis in RA.

## 1. Introduction

Rheumatoid arthritis (RA) is the most common systemic autoimmune disease affecting approximately 1% of the population worldwide. The persistent synovitis and thereby bone erosion are the hallmark of RA. Though the precise etiology of RA still remains elusive, osteoclast, formed by fusion of mononuclear precursors of the monocyte/macrophage, is the cell ultimately responsible for bone destruction in RA [1].

The past decade has witnessed a number of regulators of osteoclast differentiation and function. Among them, receptor activator of nuclear factor *κ*B ligand (RANKL) plays the most important role in osteoclast development, activity, and survival [2]. It has been previously reported that mice deficient in RANKL are protected from bone erosion in a serum transfer model of arthritis [3]. In RA patients, local and systemic increased RANKL levels are associated with bone resorption, suggesting their pivotal role in mediating bone erosion [4]. RANKL exerts its functions by binding to its unique receptor RANK, and osteoprotegerin (OPG) acts as its natural decoy receptor by blocking the RANK/RANKL interaction. Mice lacking OPG exhibit severe osteoporosis and bone erosions [5], implicating the importance of RANKL/OPG balance for maintaining osteoclast homeostasis.

Substantial evidence has suggested that proinflammatory cytokines, such as IL1*β*, TNF*α*, IL-6, and IL-17 [6–10], and some hormones, including parathyroid hormone (PTH) and 1,25-dihydroxyvitamin D_3_ (1,25(OH)_2_D_3_), are involved in regulating RANKL expression [11]. Importantly, several functional vitamin D response elements (VDREs) upstream from the transcription start site of the murine and human RANKL gene have been recently identified, which potentially make 1,25(OH)_2_D_3_ one of the most potent stimulators of RANKL expression [12, 13].

Traditionally, vitamin D was thought to maintain calcium homeostasis; however, the role of vitamin D in autoimmune disease has been a topic of much interest recently. Emerging evidence indicated that vitamin D deficiency is quite common in autoimmune disease, including systemic lupus erythematosus (SLE), RA, and other autoimmune rheumatological disorders [14–16]. In RA, the prevalence of vitamin D deficiency ranges from 30% to 63%, and serum vitamin D levels are inversely related to RA disease activity [17]. Moreover, for patients with RA, each 10 ng/mL increase of the serum 1,25(OH)_2_D_3_ level is associated with a 0.3-point decrease of the Disease Activity Score 28-joint assessment (DAS28) and a 25% decrease of C-reactive protein (CRP) levels [18], suggesting that vitamin D supplement is benefit for RA treatment and should be advocated in clinical practice.

Considering that 1,25(OH)_2_D_3_, the active form of vitamin D, has been suggested as a potent inducer of RANKL expression [12, 13], it should clarify whether vitamin D supplement could result in RANKL overexpression and thereby facilitate excessive osteoclastogenesis and bone resorption in RA. Here, we study the effect of 1,25(OH)_2_D_3_ on the regulation of RANKL/OPG axis in a simulative inflammatory context by human rheumatoid fibroblast-like synoviocyte MH7A stimulated with IL1*β* and the formation of osteoclast by osteoclast precursors RAW264.7 cell line treated with IL1*β* and 1,25(OH)_2_D_3_ combined.

## 2. Materials and Methods

### 2.1. Human Rheumatoid Fibroblast-Like Synoviocyte Cell Line MH7A and Culture

Human rheumatoid fibroblast-like synoviocyte MH7A cells used in this study were a generous gift from Dr. Seiichi Tanuma (Tokyo University of Science). MH7A cells were isolated from the intra-articular soft tissue of knee joints of RA patients and were established as a cell line by transfection with the SV40 T antigen [19]. Primary RA fibroblast-like synoviocyte (RA-FLS) samples were obtained from three RA patients after synovectomy or arthroplasty. A written consent was signed by these patients. MH7A and RA-FLS cells were cultured in DMEM medium supplemented with 10% fetal bovine serum (Gibco, Carlsbad, CA, USA), 100 U/mL penicillin, and 100 *μ*g/mL streptomycin (Sigma-Aldrich, St. Louis, MO) at 37°C in a humidified atmosphere of 5% CO_2_ in air.

### 2.2. 1,25(OH)_2_D_3_ Treatment

MH7A cells were stimulated with 20 ng/mL IL1*β* (Peprotech, NJ, USA) to induce RANKL expression and then treated with different concentrations of 1,25(OH)_2_D_3_ (0.1 nM, 1 nM, 10 nM, and 100 nM, Sigma-Aldrich, St. Louis, MO) for 48 h. The cell pellet and its supernatants were collected for further real-time PCR and ELISA analysis. Experiments were performed in triplicates from three separated studies.

### 2.3. Real-Time PCR

1,25(OH)_2_D_3_ effects upon gene expression were studied by extracting total RNA from treated cells using Trizol reagent (Invitrogen, Carlsbad, CA, USA). About 1 *μ*g of RNA was reverse transcribed using the transcript RT system. Real-time PCR (qPCR) was performed using PrimeScriptRT Master Mix (Takara Bio, Japan). Relative expressions of RANKL, its natural decoy receptor OPG and vitamin D receptor (VDR), and proinflammatory cytokines of TNFa and IL-6 in MH7A were normalized using the expression levels of *β*-actin and calculated by the 2^−ΔΔ*Ct *^ method.

### 2.4. Immunofluorescence

MH7A cells treated with 1,25(OH)_2_D_3_ and IL1*β* were cultured on cover slips for 48 hours. Then, MH7A cells were fixed in 4% paraformaldehyde for 10 mins and permeabilized with 0.3% Triton X-100 in PBS for 5 mins. Cells were labeled with the following antibodies: anti-human RANKL antibody (Abcam, Cambridge, UK), TRITC-Conjugated AffiniPure Goat Anti-Rabbit IgG (ZSGB-BIO; Beijing, China). The incubation conditions for the primary, secondary antibody, and 6-diamidine-20-phenylindole dihydrochloride (DAPI) were 14 to 18 h at 4°C, 100 mins, and 2 mins at room temperature, respectively. Images were acquired and processed digitally under a fluorescence microscope (Nikon, Tokyo, Japan).

### 2.5. Osteoclastogenesis

Murine monocytic RAW264.7 cells were cultured in 12-well dishes at a density of 1 × 10^4^. RAW264.7 were treated with M-CSF (20 ng/mL) and RANKL (50 ng/mL) for 3 days. At 3 days pretreatment, RAW264.7 cells were washed three times with PBS (phosphate-buffered saline) and then incubated with IL1*β* (20 ng/mL) in the presence or absence of 1,25(OH)_2_D_3_ (10 nM) or 1,25(OH)_2_D_3_ alone for 5 days. Osteoclast formation was assessed by counting the total number of multinucleated (>3 nuclei) tartrate-resistant acid phosphatase (TRAP) positive cells present per well at day 8.

### 2.6. Cytokine Production

Cytokine production was determined in cell culture supernatants using ELISA specific for human IL-6 and TNF*α* (BOSTER-BIO; Wuhan; China) following manufacturer's guidelines.

### 2.7. Statistical Analysis

Results were expressed as mean ± standard deviation and assessed for the difference using ANOVA test. Results are representative of three separate experiments. Statistical analyses were performed by SPSS version 18.0 software (SPSS, Chicago IL, USA). *P* < 0.05 was considered as significant. 

## 3. Results

### 3.1. Effect of 1,25(OH)_2_D_3_ on RANKL Expression in MH7A

**Figure 1:**
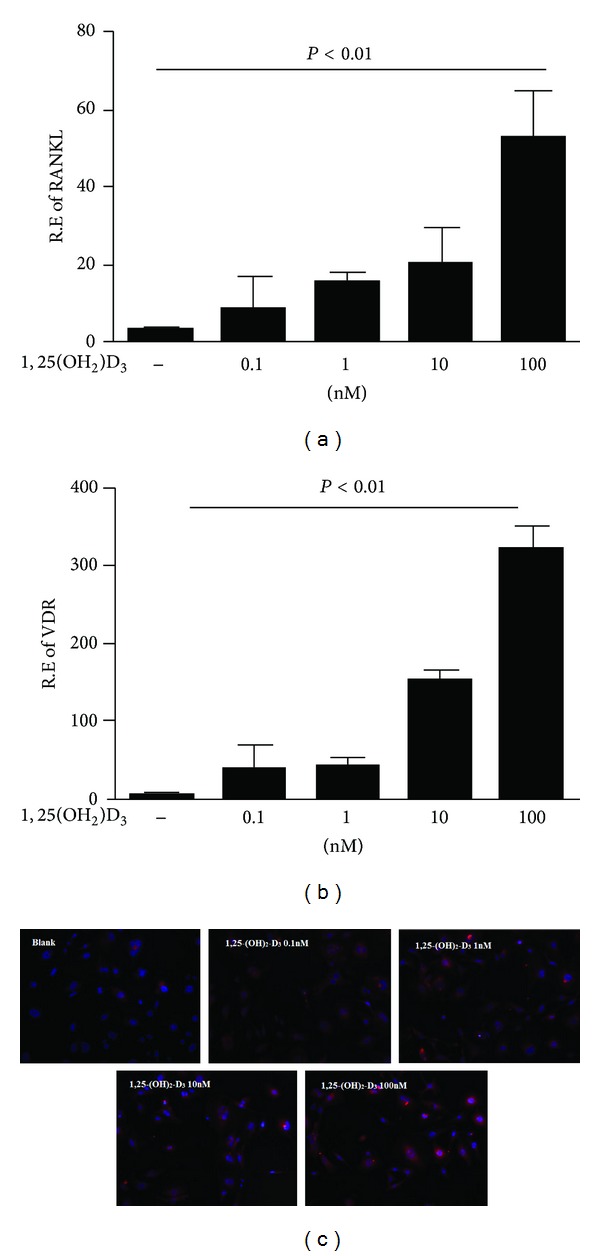
The effect of 1,25(OH)_2_D_3_ on RANKL and VDR expression in fibroblast-like synoviocyte MH7A cells. MH7A cells were treated with different concentrations of 1,25(OH)_2_D_3_ (0.1 nM, 1 nM, 10 nM, and 100 nM) for 48 h. RANKL (a) and VDR (b) mRNA expression were analyzed by real-time PCR. The presence of RANKL staining cells (marked with red) in cultured MH7A was detected by immunofluorescence (c).

Given that 1,25(OH)_2_D_3_ has been suggested as a potent inducer of RANKL expression in stromal and osteoblastic cells, we first validated the effect of 1,25(OH)_2_D_3_ on RANKL expression in fibroblast-like synoviocyte MH7A cells. We also found that 1,25(OH)_2_D_3_ could increase proliferative activity of MH7A (data not shown). MH7A cells were treated with different concentrations of 1,25(OH)_2_D_3_ for 48 h, and then transcript levels of RANKL were analyzed by real-time PCR. As illustrated in Figure 1(a), RANKL expression was significantly increased in a dose-dependent manner in MH7A upon 1,25(OH)_2_D_3_ stimulation (Figure 1(a)). Immunofluorescence also indicated that the amount of RANKL staining cells among cultured MH7A elevated after treatment with 1,25(OH)_2_D_3_ (Figure 1(c)). In addition, we found that 1,25(OH)_2_D_3_ could markedly enhance the expression of VDR in MH7A (*P* < 0.01), suggesting that the responsiveness to 1,25(OH)_2_D_3_ stimulation might be via activation the specific receptor of vitamin D in synoviocyte (Figure 1(b)).

### 3.2. Effect of 1,25(OH)_2_D_3_ on IL*β*-Induced RANKL/OPG Expression in MH7A

**Figure 2:**
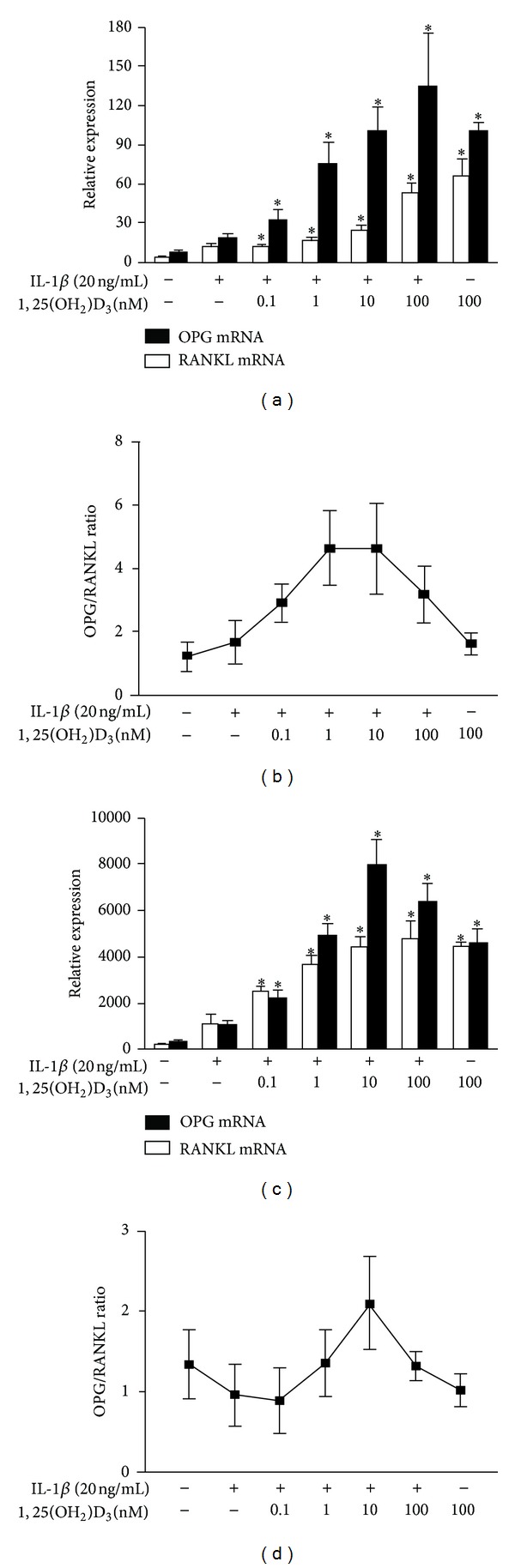
Effect of 1,25(OH)_2_D_3_ on IL*β*-induced RANKL and OPG expression in MH7A and primary RA fibroblast-like synoviocyte. MH7A cells were stimulated with 20 ng/mL IL1*β* and then treated with different concentrations of 1,25(OH)_2_D_3_ (0.1 nM, 1 nM, 10 nM, and 100 nM) for 48 h.The effect of 1,25(OH)_2_D_3_ on RANKL and OPG expression in IL1*β*-induced MH7A (a) and RA-FLS (c) was analyzed by real-time PCR.The alteration of OPG/RANKL ratio in IL1*β*-induced MH7A (b) and RA-FLS (d) was analyzed (b). The data points shown are the mean ± SD for three independent experiments, each in triplicate. *P < 0.05 compared to cells cultured with IL-1*β* alone.

In the pathological condition of RA, synovial fibroblasts in the inflamed joints could cause the exaggerated expression of multiple proinflammatory cytokines including TNF*α*, IL6, IL-17, and IL1*β*, all of which result in increased local joint RANKL expression. Given previous study reported that only IL1*β* or IL-6 plus soluble IL-6 receptor (but not IL-6 alone) could show the ability to induce RANKL expression in vitro after RA fibroblast-like synoviocyte treatment with TNF*α*, IL-6, IL-17, and IL1*β* [20], we stimulated MH7A cells with IL1*β* in current study. As expected, expression levels of RANKL and OPG increased in MH7A upon IL1*β* stimulation (Figure 2(a)). Furthermore, 1,25(OH)_2_D_3_ treatment significantly enhanced expression of RANKL and OPG at a dose-dependent manner in IL1*β*-induced MH7A cells compared with those without 1,25(OH)_2_D_3_ treatment (*P* < 0.05) (Figure 2(a)). Our data suggested that 1,25(OH)_2_D_3_ and IL*β* could synergistically prompt expression of RANKL and OPG in MH7A.

Moreover, it seems that 1,25(OH)_2_D_3_ tends to induce a more robustly increased expression of OPG than RANKL, which eventuallyleadsto the significantly increased ratio of OPG/RANKL in IL1*β*-stimulated MH7A (Figure 2(b)). The groups treated with 1 nM or 10 nM 1,25(OH)_2_D_3_ conferred about 5-fold increased OPG/RANKL ratio in IL1*β*-stimulated MH7A compared with the nontreatment group (Figure 2(b)). 

We further confirmed our data in primary RA fibroblast-like synoviocyte. Similar to the result presented in MH7A cell lines, a significantly elevated OPG and RANKL expression and OPG/RANKL ratiowereobserved in IL1*β*-induced primary RA-FLS in the presence of 1,25(OH)_2_D_3_ compared with thosein theabsence of it (Figures 2(c) and 2(d)).

### 3.3. Effect of 1,25(OH)_2_D_3_ on Osteoclastogenesis in the Presence of IL1*β* in Osteoclast Precursors RAW264.7 Cell Line

**Figure 3:**
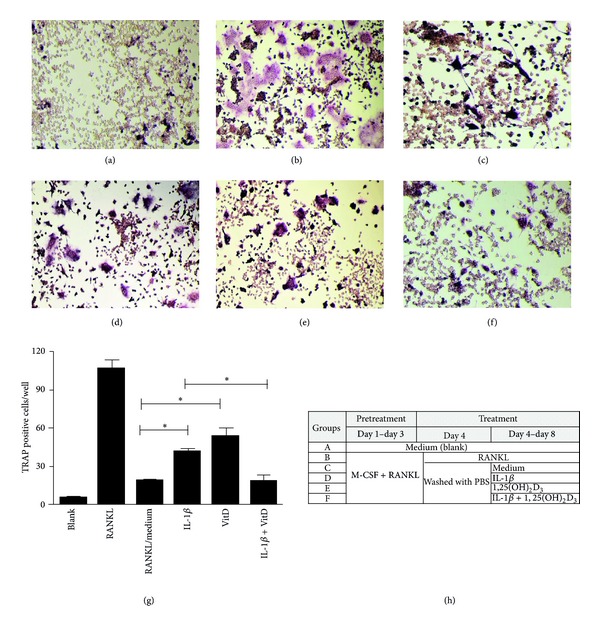
Effect of 1,25(OH)_2_D_3_ on osteoclastogenesis in the presence of IL1*β* in osteoclast precursors RAW264.7 cell line. RAW264.7 were treated with M-CSF (20 ng/mL) and RANKL (50 ng/mL) for 3 days. At 3 days pretreatment, cells were washed three times with PBS and were then incubated with IL1*β* (20 ng/mL) in the presence or absence of 1,25(OH)_2_D_3_ (10 nM) or 1,25(OH)_2_D_3_ alone for 5 days. TRAP positive cells with numerous (>3) unstained nuclei were considered as mature osteoclasts. RAW264.7 treated with medium as negative control was shown in (a). TRAP positive cells in unwashed RAW264.7 treated with RANKL continually were shown in (b) and in washed RAW264.7 cells treated with medium were shown in (c). After washing, TRAP positive cells in RAW264.7 incubated with IL1*β*, 1,25(OH)_2_D_3_ or IL1*β* plus 1,25(OH)_2_D_3_ were shown in ((d)–(f)). TRAP positive cells number in different groups were shown in (g) (*P < 0.05). The diagram of treatment process in RAW264.7 was shown in (h).

Given 1,25(OH)_2_D_3_ is capable of increasing OPG/RANKL ratio in MH7A after IL1*β* stimulation, we next investigated whether the presence of 1,25(OH)_2_D_3_ or IL1*β* could affect osteoclastogenesis in osteoclast precursors RAW264.7 cell line. RAW264.7 cells were pretreated with M-CSF and RANKL for 3 days. Then, RAW264.7 cells were washed three times with PBS and incubated with IL1*β* in the presence or absence of 1,25(OH)_2_D_3_ (10 nM) for 5 days. Unwashed RAW264.7 cells were treated with RANKL as a positive control. As expected, the presence of RANKL in medium (Figure 3(b)) significantly stimulated osteoclast formation. After washing out RANKL in medium, either IL1*β* (Figure 3(d)) or 1,25(OH)_2_D_3_ (Figure 3(e)) treatment could maintain modest osteoclast formation compared to cells without treatment (Figure 3(c)). However, TRAP positive cells were obviously decreased whencells were treated with 1,25(OH)_2_D_3_ and IL1*β* combined(Figure 3(f)).

### 3.4. Effect of 1,25(OH)_2_D_3_ on IL*β*-Induced TNF*α* and IL-6 Production in MH7A

**Figure 4:**
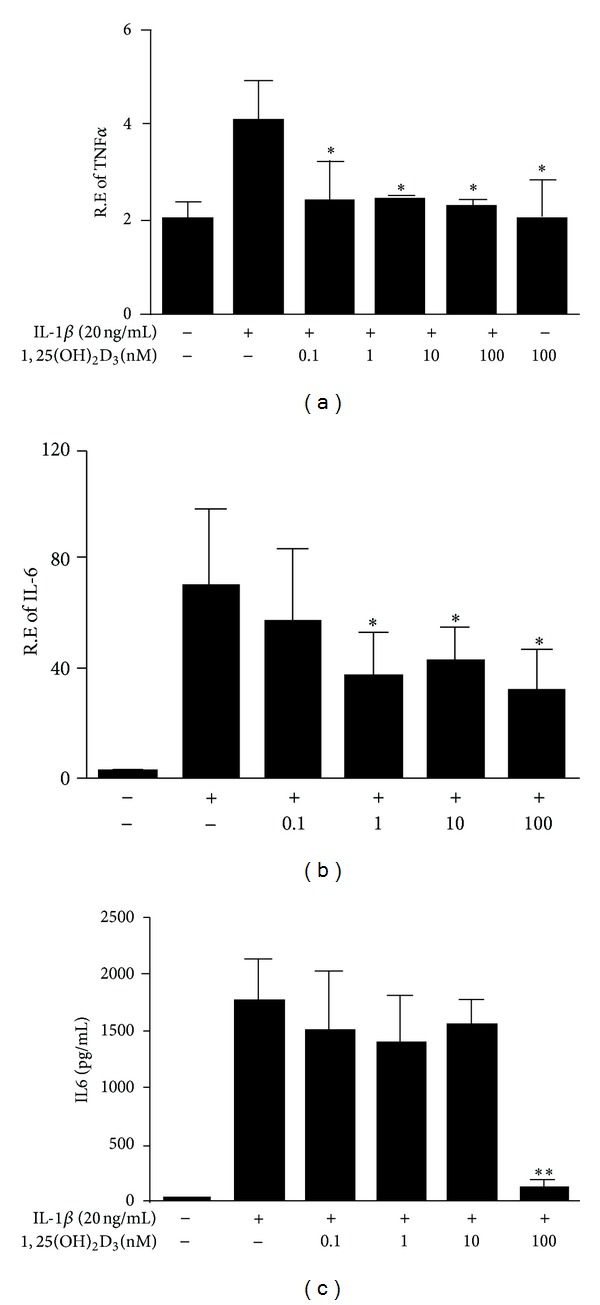
Effect of 1,25(OH)_2_D_3_ on IL*β*-induced TNF*α* and IL-6 production in MH7A. MH7A cells were stimulated with 20 ng/mL IL1*β* and then treated with different concentrations of 1,25(OH)_2_D_3_ (0.1 nM, 1 nM, 10 nM, and 100 nM) for 48 h. The effects of 1,25(OH)_2_D_3_ on IL*β*-induced TNF*α* (a) and IL-6 (b) mRNA expression in MH7A was analyzed by real-time PCR, and the production of IL-6 in supernatants was shown in (c). The data shown are the mean ± SD for three independent experiments, each in triplicate. *P < 0.05 compared to cells cultured with IL-1*β* alone. **P < 0.01 compared to cells cultured with IL-1*β* alone.

We next evaluated potential impact of 1,25(OH)_2_D_3_ on the production of proinflammatory cytokines in MH7A. Expression levels of TNF*α* and IL-6 significantly increased in MH7A cells after stimulation with IL-1*β* for 48 hours (Figures 4(a)–4(c)). However, transcript levels of TNF*α* (Figure 4(a)) and IL-6 (Figure 4(b)) in IL-1*β*-stimulated MH7A cells significantly reduced after treatment with 1,25(OH)_2_D_3_ at the dosage of 0.1 to 100 nM and 1 to 100 nM, respectively, compared with those not treated with 1,25(OH)_2_D_3_ (Figure 4(b)). Protein levels of TNF*α* and IL-6 in supernatants were analyzed by ELISA. Treatment with 1,25(OH)_2_D_3_ at the dosage of 100 nM significantly inhibited IL-6 production in supernatants of IL-1*β*-stimulated MH7A cells (Figure 4(c)). Unfortunately, we failed to accurately measure TNF*α* levels in supernatants which might be due to the relative low concentration or there is a dichotomy between mRNA and protein expression for TNF*α* (data not shown). In addition, we tried to explore the role of 1,25(OH)_2_D_3_ on the expression of IL-17, another important proinflammatory cytokine in RA. However, there were no changes in IL-17 levels after MH7A challenge with IL*β* (data not shown). Taken together, these data indicated that 1,25(OH)_2_D_3_ had favorable effects on preventing proinflammatory cytokines production in MH7A upon IL-*β* stimulation.

## 4. Discussions

Since some vitamin D responsive elements (VDREs) have been identified on murine and human RANKL promoter in previous studies [12, 13, 21], it is therefore not surprising that 1,25(OH)_2_D_3_, the physiologically active metabolite of vitamin D, is a potent modulator of RANKL expression [5]. The strong link between RANKL and osteoclastogenesis naturally makes us suspect whether vitamin D supplementation may result in RANKL overexpression and thereby accelerate osteoclast-mediated bone resorbing in RA. However, the inhibitory effect of 1,25(OH)_2_D_3_ on the progression of arthritis in murine experimental models and RA patients [17, 22, 23] suggested a possible therapeutic efficacy of vitamin D supplementation in RA. Here, our data provided the lines of evidence to support the therapeutic role of vitamin D in RA by in vitro studies.

The biologic effect of 1,25(OH)_2_D_3_ is mediated through the vitamin D receptor (VDR). Thus, we first validated the expression of VDR in MH7A cells and also found 1,25(OH)_2_D_3_ could increase proliferative activity of MH7A, indicating a responsive ability of MH7A to vitamin D stimulation. As expected, we confirmed what was previously reported that 1,25(OH)_2_D_3_ is a potent inducer of RANKL expression in MH7A [5, 24]. Considering that chronic synovial inflammation is a hallmark of RA, the real modulatory effect of 1,25(OH)_2_D_3_ on RANKL should be deliberated under the inflammatory microenvironment of RA.

RANKL is produced by a number of different cell types including T cells, B cells, dendritic cells, macrophages, and synovial fibroblasts in RA [6–10]. Substantial evidence has suggested that proinflammatory cytokines such as IL1*β*, TNF*α*, IL-6, IL-17, and others, derived from synovial fibroblasts in the inflamed joints, are the primary trigger for the local or systemic high expression of RANKL in RA [25, 26], which is the main explanation of inflammation-induced osteoclast activation and bone lose. Giventhatinflamed synoviocytes are one of the main sources of proinflammatory cytokines production in RA [27, 28], here, synoviocyte MH7A were stimulated with IL-1*β*. Our data demonstrated that 1,25(OH)_2_D_3_ could decrease TNF-a and IL-6 production induced by IL*β* in MH7A, confirming the previously reported anti-inflammatory action of 1,25(OH)_2_D_3_ [29]. 

We observed that expression levels of RANKL and OPG significantly increased in MH7A after IL-1*β* treatment. Specifically, compared with the relative modest RANKL expression, it seems that 1,25(OH)_2_D_3_ tends to induce a more robust OPG expression in IL1*β* stimulated MH7A, which result in a significant increased ratio of OPG/RANKL. OPG acts as an inhibitor of osteoclast formation and the elevated OPG/RANKL ratio may represent a low state of osteoclastogenesis [30]. We next proved osteoclastogenesis was obviously decreased when osteoclast precursors RAW264.7 cell line were treated with both 1,25(OH)_2_D_3_ and IL1*β*. It is possible that, although 1,25(OH)_2_D_3_ is capable of inducing RANKL expression by interacting with VDREs in the RANKL promoter in the physiological state, upregulation OPG/RANKL ratio and accordingly suppressing inflammation-induced osteoclastogenesis might support the protective role of 1,25(OH)_2_D_3_ in arthritis models and RA patients.

In addition, the immunomodulatory role of vitamin D on both innate and adaptive immune system might be another explanation for the benefit of vitamin D supplementation in RA and arthritis models. Vitamin D could downregulate Th1-dependent responses [31] by suppressing IL-17A and IFN-*γ* production and stimulating IL-4 and IL-10 production in RA [32]. Moreover, 1,25(OH)_2_D_3_ directly modulated human Th17 polarization, accompanied by reducing the production of Th17 cytokines IL-17A, IL-17 F, and IL-22 in early RA [33]. 

In summary, althoughithas a biological function to induce RANKL expression, 1,25(OH)_2_D_3_ could upregulate OPG/RANKL ratio and mediate anti-inflammatory action in an inflammatory milieu of synoviocyte, contributing to inhibit inflammation-induced osteoclastogenesis in RA. These preliminary results are encouraging, and further clinical study is needed to confirm the potential role of vitamin D supplementation in daily clinical practice.

## Abbreviations

1,25(OH)_2_D_3_: 1,25-Dihydroxyvitamin D_3_
IFN*γ*: Interferon gamma
IL: Interleukin
OPG: Osteoprotegerin
RA: Rheumatoid arthritis
RANKL: Receptor activator of nuclear factor *κ*B ligand
SLE: Systemic lupus erythematosus
TNF*α*: Tumor necrosis factor alpha
VDR: Vitamin D receptor
VDREs: Vitamin D response elements.
